# Characterization and virulence of *Streptococcus agalactiae* deficient in *SaeRS* of the two-component system

**DOI:** 10.3389/fmicb.2023.1121621

**Published:** 2023-04-17

**Authors:** Shiyu Li, Wei Li, Qiancai Liang, Jizhen Cao, Han Li, Zhicheng Li, Anxing Li

**Affiliations:** ^1^State Key Laboratory of Biocontrol/Guangdong Provincial Key Laboratory of Improved Variety Reproduction in Aquatic Economic Animals and Institute of Aquatic Economic Animals, School of Life Sciences, Sun Yat-sen University, Guangzhou, China; ^2^Innovative Institute of Animal Healthy Breeding, College of Animal Sciences and Technology, Zhongkai University of Agriculture and Engineering, Guangzhou, China; ^3^Agricultural Technology Promotion Center of Maoming City, Maoming, China

**Keywords:** *Streptococcus agalactiae*, two-component system, *SaeRS*, virulence, colonization

## Abstract

There are a variety of regulatory systems in bacteria, among which the two-component system (TCS) can sense external environmental changes and make a series of physiological and biochemical reactions, which is crucial for the life activities of bacteria. As a member of TCS, *SaeRS* is considered to be an important virulence factor in *Staphylococcus aureu*s, but its function in tilapia (*Oreochromis niloticus*)-derived *Streptococcus agalactiae* remains unknown. To explore the role of *SaeRS* in regulating virulence in the two-component system (TCS) of *S. agalactiae* from tilapia, Δ*SaeRS* mutant strain and CΔ*SaeRS* complementary strain were constructed by homologous recombination. The results showed that the abilities of growth and biofilm formation of Δ*SaeRS* strain were significantly decreased when cultured in a brain heart infusion (BHI) medium (*P* < 0.01). Also, the survival rate of the Δ*SaeRS* strain in blood was decreased when compared with the wild strain *S. agalactiae* THN0901. Under the higher infection dose, the accumulative mortality of tilapia caused by the Δ*SaeRS* strain was significantly decreased (23.3%), of which THN0901 and CΔ*SaeRS* strains were 73.3%. The results of competition experiments in tilapia showed that the invasion and colonization abilities of the Δ*SaeRS* strain were also dramatically lower than those of the wild strain (*P* < 0.01). Compared with the THN0901, the mRNA expression levels of virulence factors (*fbsB*, *sip*, *cylE*, *bca*, etc.) in the Δ*SaeRS* strain were significantly down-regulated (*P* < 0.01). *SaeRS* is one of the virulence factors of *S. agalactiae*. It plays a role in promoting host colonization and achieving immune evasion during the infection of tilapia, which provides a basis for exploring the pathogenic mechanism of *S. agalactiae* infected with tilapia.

## 1. Introduction

*Streptococcus agalactiae*, or group B *Streptococcus* (GBS), is a major bacterial species of the genus *Streptococcus* (Tavares et al., 2019), which can cause invasive infection in a range of hosts, such as humans (Furfaro et al., 2018), cattle (Lyhs et al., 2016), and fish (Boonyayatra et al., 2020). GBS infection of tilapia (*Oreochromis niloticus*) is associated with septicemia and meningoencephalitis (Evans et al., 2008). In addition, GBS can cause enormous economic losses to the tilapia industry (Liu et al., 2016). For example, outbreaks in which up to 30% mortality in tilapia culture have been reported in Thailand (Naraid et al., 2008; Kayansamruaj et al., 2014) and many other countries (Anshary et al., 2014; Aisyhah et al., 2015). The exact and complete pathogenic mechanism of GBS on tilapia has not been clarified, so it is necessary to explore its virulence factors. The two-component system (TCS) in bacteria is used to make a series of responses for adaptive regulation in environment changes (Groisman, 2016), and is consisted of two main components, a histidine kinase and a response regulator (Capra and Laub, 2012; Christopher et al., 2016). Histidine kinase can identify specific stimulus signals and activate response regulators through auto-phosphorylation and participate in gene transcription, resulting in adaptation to the particular environment by binding to specific DNA sequences (Mitrophanov and Groisman, 2008). TCS is required for bacterial growth and fitness (Mitrophanov and Groisman, 2008) and drug resistance (Tierney and Rather, 2019) and is also associated with the induction of virulence (Dagmar and Roy, 2006), and biofilm-forming ability (Badal et al., 2020). A previous study demonstrated that inactivation of the *SaeRS* system in *Staphylococcus aureus* significantly decreased apoptosis or death of lung epithelial cells (A549), and attenuated virulence in a murine infection model (Liang et al., 2006). A previous study in *Staphylococcus epidermidis* showed that the deletion of *SaeRS* affected the expression levels of genes with a variety of functions, including bacterial autolysis (*lrgA, arlR*, and *lytS*), biofilm formation (*ebhA*), leucine biosynthesis (*leuD*), protein hydrolysis (*clpP*), stress resistance (*asp23*), and cell viability (*yycH*) (Lou et al., 2011).

In *S. agalactiae* strains isolated from humans, plasminogen binding protein (*pbsP*) and other factors can promote host colonization of GBS through the up-regulation of *SaeRS* in TCS by the mouse vaginal colonization model (Cook et al., 2018). In *S. aureus*, *SaeRS* plays an important role in the regulation of virulence factors and pathogenicity. Such as regulation of relevant virulence factors α-Hemolysin, lipase, coagulase and adhesin, etc., (Ericson et al., 2017; Liu et al., 2020, 2021). Studies on *S. epidermidis* showed that the deletion of *SaeRS* would affect genes with multiple functions, including bacterial autolysis (*lrgA*, *arlR*, and *lytS*), biofilm formation (*ebhA*), leucine biosynthesis (*leuD*), protein hydrolysis (*clpP*), stress resistance (*asp23*) and cell viability (*yycH*) (Lou et al., 2011).

*SaeR*S plays a key role in the pathogenicity of *S. agalactiae.* However, the characteristics and virulence of GBS affecting tilapia are still unknown. To study the role of *SaeRS* in GBS, a Δ*SaeRS* mutant was constructed with deletion of the genes that encode both the histidine kinase (*SaeS*) and the response regulator (*SaeR*) and its corresponding complementary strain CΔ*SaeRS*, and the role of the gene deletion mutant was assessed under *in vivo and in vitro* conditions.

## 2. Materials and methods

### 2.1. Bacteria and animals

*Streptococcus agalactiae* THN0901, a wild-type virulent strain, was isolated from tilapia infected with GBS obtained from an intensive tilapia farm, Hainan, China. GBS strains were cultured aerobically overnight at 28°C, 180 rpm in a brain heart infusion (BHI) bath and inoculated into a BHI medium with a diluted rate of 1:100 (vol/vol) for 12 h under the same conditions. Our laboratory has previously sequenced the whole genome of THN0901, so the primer design in this experiment is derived from the genome of THN0901. Tilapia (25 ± 5 g of body weight) were purchased from a farm in Guangzhou, Guangdong, China, which were acclimated with flowing water at 28 ± 0.5°C for 2 weeks and fed with commercial feed twice a day (1% body weight every time). All protocols involved in the care of fish were approved by the Institutional Animal Care and Use Committees at Sun Yat-sen University (SYSU-IACUC-2020-B0740).

### 2.2. Strain construction

**FIGURE 1:**
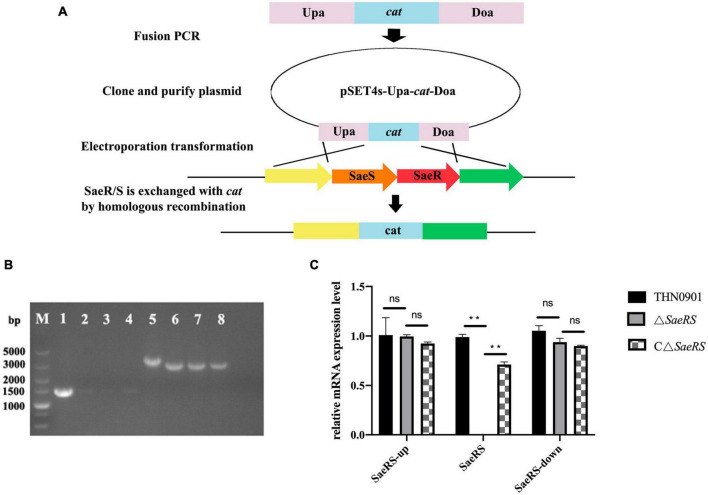
**(A)** Construction of the *SaeRS* gene deletion strain of *S. agalactiae* THN0901 by homologous recombination. **(B)** Detection of the Δ*SaeRS* mutant by the polymerase chain reaction (PCR). Lanes 1–4 using the primers Sae-F and Sae-R; Lanes 5–8 using the primers Sae-up-F and Sae-down-R; The primer sources used in lanes 1–4 are upstream homologous arm and downstream homologous arm. The primer sources used in lanes 5–8 are WP-000703870.1 and AK1948 13.1. Lane M is DL5000 DNA marker; Lane 1 and 5 is *S. agalactiae* THN0901, Lane 2 and 6 is 5th-generation Δ*SaeRS*; Lane 3, and 7 is 10th-generation Δ*SaeRS*, Lane 4 and 8 is 15th-generation Δ*SaeRS*, respectively. **(C)** Relative messenger RNA (mRNA) expression level of gene expression up and down stream of *SaeRS* among three strains. Each experiment was performed in triplicate, and data were presented as mean ± standard deviation (SD), *: 0.01 < *P* < 0.05, **: *P* < 0.01.

To generate a functional deletion mutant of *SaeRS*, a 1,257-bp fragment upstream of *SaeRS* and a downstream homologous sequence (1,042 bp) from *S. agalactiae* THN0901 strain were amplified with the primers Sae-up-F/R, Sae-down-F/R, and cat-F/R (Table 1). The chloramphenicol resistant gene (*cat*) (1,056 bp) was acquired from the plasmid pSET5s using the primers cat-F and cat-R (Table 2). The amplified products were cloned into pMD-19T plasmid to construct recombinant fragment Upa-*cat*-Doa replacing the sequence of *SaeRS* with *cat* gene, which was ligated with cleaved thermo-sensitive suicide vector pSET4s (Miaoling Biological Co., Ltd., China) showing spectinomycin resistance (Figure 1A). The correct orientation insert and sequence of pSET4s-Upa-*cat*-Doa plasmid were confirmed by DNA sequencing and then transformed into THN0901 wild strain *via* electroporation (Takamatsu et al., 2001). The proposed strains were selected at 28°C and cultured at 37°C in a BHI bath with chloramphenicol (10 μg/mL) several times to contribute to homologous recombination. The mutant strain Δ*SaeRS* was achieved by selection for sensitive to chloramphenicol resistance (Figure 1B), and it was confirmed by DNA sequencing and quantitative polymerase chain reaction (qPCR) analysis using the *SaeRS* gene-specific primers (Table 1). Using the sae-HB-F and sae-HB-R primers, the *SaeRS* and its upstream promoter sequence were linked together into the pSET2s expression vector to construct a complementing expression vector, which was electroporated into the Δ*SaeRS* mutant strain and verified by PCR, sequencing and quantitative reverse transcription PCR (RT-qPCR) to get the complementary strain (Figure 1C).

**TABLE 1:** List of primers mutant strain validation.

Name	Primers (5′–3′)
Sae-up-F	CCGGAATTCGGTGGAGATGGCATGTTAA
Sae-down-R	CCCAAGCTTATTGGATGATCAGTCATCGT
Sae-F	AGAAGCACTCAAAGAGGTAG
Sae-R	TTATCCCATAGCCTTGGTC
Sae-up-qp-F	CCTTAAAGCTTCAGAGGGAGCA
Sae-up-qp-R	CCTGTCGCAAAGGCTAGACTAA
Sae-qp-F	CAGACACGGCTGAACCTCAT
Sae-qp-R	CGAAACATCTGGCGCAACAT
Sae-down-qp-F	CCTTAAAGCTTCAGAGGGAGCA
Sae-down-qp-R	CCTGTCGCAAAGGCTAGACTAA
Cat-F	AGACGTTATCTAGAGCAAGCCGGGATCCGCACCGAACT AGAGCTTGATGA
Cat-R	CATTCAGTTGATGTGCAAGTTGCACTGCAGTGCATAAT TCGATGGGTTCC
Sae-HB-F	CGCGGATCCGCAAATGTCATCACAAGCC
Sae-HB-R	AACTGCAGGTTGGCAGTTCCTTACAATC

The underline is the restriction site.

**TABLE 2:** Cumulative survival of tilapia challenged.

Bacterial strain	Infection dose (CFU/fish)	Total fish	Survival fish	Motility rate
THN0901	3.5 × 10^8^	30	8	73.3%
	3.5 × 10^7^	30	14	53.3%
	3.5 × 10^6^	30	20	33.3%
Δ*SaeRS*	3.5 × 10^8^	30	23	23.3%
	3.5 × 10^7^	30	27	10%
	3.5 × 10^6^	30	28	6.7%
CΔ*SaeRS*	3.5 × 10^8^	30	8	73.3%
	3.5 × 10^7^	30	14	53.3%
	3.5 × 10^6^	30	16	46.7%

### 2.3. Growth analysis

The wild strain THN0901, the mutant strain Δ*SaeRS* and complementary strain CΔ*SaeRS* were cultured to analyze the bacterial growth characteristics. Proliferation was assessed in BHI liquid medium and strains were first cultured at 28°C overnight and then inoculate into 100 mL fresh BHI medium at a ratio of 1/50 (vol/vol) by shaking at 180 rpm. It is worth mentioning that the mutant and complementary strains were always cultured in the presence of antibiotics. The optical density at 600 nm (OD_600_) value of the culture was measured in 1-h intervals to describe the growth curve of the above strains.

### 2.4. Determination of biofilm formation

The biofilm formation of GBS was quantified as described by a previous study (O’Toole and Kolter, 1998) with slight modification. First, fresh overnight cultures of *S. agalactiae* wild strain THN0901, mutant strain Δ*SaeRS* and complementary strain CΔ*SaeRS* were taken and adjusted to OD_600_ = 1.0 by adding BHI medium, and then 200 μL was added to each well of a 96-well plate. A fresh BHI medium was used as a negative control. The supernatant of the wells was carefully discarded and washed twice with 1 × phosphate-buffered saline (PBS) to remove non-adherent cells. Then, the biofilm-forming ability was assessed by staining with 50 μL crystal violet at 0.1% for 20 min at room temperature. The supernatant was discarded and washed twice. The plate was dried at room temperature, and 100 μL of ethanol: acetone (8:2) was added to each well. The absorbance at 575 nm (A_575_) was measured. Each experiment was performed in four independent experiments. Two-tailed Student’s *t*-test was performed to analyze the mean ± standard deviation (SD).

### 2.5. Mixed competition experiments *in vitro* and *in vivo*

For *in vitro* experiments, the overnight cultures of THN0901 strain and Δ*SaeRS* mutant strain were adjusted to the same OD value, and then combined in equal volume in a microcentrifuge tube, which was diluted in 50 mL of BHI medium at 1:100 (vol/vol). Diluted and incubated aerobically at 28°C in a shaker. Serially diluted bacterial solutions were then plated onto BHI plates after 2, 4, 6, 9, 12, and 24 h (Patterson et al., 2012). The competition index (CI), the ratio of the Δ*SaeRS* mutant to the total bacterial count (TBC), was determined by counting the TBC of the samples on the BHI plate and identifying the bacteria on the BHI plate with 20 μg/mL chloramphenicol. For *in vivo* experiment, the concentration is 1.0 × 107 (CFU)/mL mutant and 1.0 × 107 CFU/mL of wild strains were mixed in equal volume, and a total of 20 Nile tilapia were intraperitoneally injected with the same amount of mixed bacterial solution (100 μL per fish). After 3, 6, and 9 h, the brain, liver, and spleen were aseptically removed from the tilapia. Ten-fold serial dilutions of whole tissue and blood samples were prepared in PBS and then plated onto BHI plates to determine CI values. The CI value was calculated in the same way as done for *in vitro* experiments. After counting, we dissected the spleen of tilapia and used the plate with antibiotics to verify the accuracy of the mutant strain. Each experiment was performed in three independent experiments. Two-tailed Student’s *t*-test was performed to analyze the mean ± SE. The null hypothesis was defined as follows: mean index is not significantly different from 1.0 (*: 0.01 < *P* < 0.05, ^**^: *P* < 0.01) (Macho et al., 2010).

### 2.6. Challenge experiment

A total of 300 fish were equally divided into 10 groups and then cultured under laboratory conditions in flowing water at 30°C. Nine experimental groups were injected intraperitoneally with THN0901, Δ*SaeRS* and CΔ*SaeRS* strains at doses of 3.5 × 10^8^, 3.5 × 10^7^, and 3.5 × 10^6^ CFU per fish, respectively. The control group was injected with sterile PBS (pH = 7.4). Mortality of challenged fish was recorded daily for 2 weeks. The mortality rate is the number of fish killed divided by the total number of fish.

### 2.7. Whole blood-killing assays

The whole blood killing method in this study refers to a previous study (Yao et al., 2015). Collect blood from tail vein of tilapia with anticoagulant tube. Fresh bacterial liquid of THN0901, Δ*SaeRS*, and CΔ*SaeRS* strains with OD_600_ = 0.4 was collected and diluted 10 times with sterile PBS. Then, 10 μL of the above dilutent was added, mixed with 300 μL of tilapia blood, incubated in a water bath at 30°C for 1 h, serially diluted, and then spread on BHI plates. Each experiment was repeated three times. After overnight culture at 37°C, the survival index was calculated as follows: the ratio of the number of colonies at the end to the number of colonies at the beginning.

### 2.8. Real-time fluorescence quantitative PCR

Total RNA was isolated from *S. agalactiae* THN0901, Δ*SaeRS*, and CΔ*SaeRS* strains using the Trizol reagent, and complementary DNA (cDNA) was synthesized using Evo M-MLV RT Kit (Vazyme, Nanjing, China) following the manufacturer’s protocol. The primers used in the study (Table 3) were designed using Primer 5.0. PCR conditions for SYBR Green RT-PCR were as follows: 95°C for 3 min, followed by 40 cycles of 95°C for 5 s and 60°C for 30 s. Melting curve analysis was performed at the end of the RT-qPCR cycle to confirm the PCR specificity. Each group had three replications, and each sample was amplified in triplicate. The expression level of the target gene relative to the *16s rRNA* gene was quantified by the 2^–ΔΔ*Ct*^ method. The RT-qPCR data were analyzed using a one-way analysis of variance (ANOVA).

**TABLE 3:** Primers for *S. agalactiae* virulence factors.

Name	Primers (5′–3′)	GeneBank ID
16srRNA-F	TTATGACCTGGGCTACAC	NP_687037.1
16srRNA-R	CCTACAATCCGAACTGAGA	
fbsB-F	AGTTGCGCAAACTTCTGTCC	AM050622.1
fbsB-R	TTTCCGCAGTTGTTACACCG	
bibA-F	CAAGCTCATCAACTTGACTCTTA	CP003810.1
bibA-R	TAGGCACATGGCTCAAAATGACG	
pavA-F	TACGGAAAATACAATCACCTACC	CHZE01000013.1
pavA-R	GCTTATGTTGTTTATCATGTGCGCG	
Srr-1-F	TCATTCCCAGTTTTATCGCTTGC	AY669067.1
Srr-1-R	TCGGAGTTACAGACTTCCAAAAT	
bac-F	TTGGACAAGCAGTATTTACATCA	AY672152.1
bac-R	ACTCTTTCGTCGTTACTTCCTTG	
sodA-F	TCAACTGCCAATCAAGATACTCC	DQ232581.1
sodA-R	GCTTTGATGTAGTTAGGACGAACA	
sip-F	AATTCAGTACATACCGTGCGGGAGA	HQ878436.1
sip-R	GTTATTTGCTGCCATATTTTGTG	
cpsD-F	GTAGGTCGTAATGGTAGGAT	NC_021195.1
cpsD-R	TTCTAGGATCATCGTCTAACTT	
cpsG-F	TAGAGATTTGATTGGGTCAGA	KC290918.1
cpsG-R	AGTTACCACTGTCATAGGAAT	
cylE-F	ATTCTCCTCCTGGCAAAGCC	AF157015.2
cylE-R	TGACGCTTGGTAGTTGCTGT	
bca-F	AATACTATGGGGATGTTTCTCAG	M97256.1
bca-R	TAACTTCTTCAATCTTATCCCTC	
hylB-F	GAATAACTACTTCACTGACGCTG	JN247784.1
hylB-R	AACGCGCCCCATATCTACTA	
cfb-F	TAGCTTAGTTATCCCAAATCCC	HQ148672.1
cfb-R	TAAAGACTTCATTGCGTGCC	
ponA-F	GCTCCTGATGAAAACTTTGTCGG	KX078639.1
ponA-R	AGAGCCCTTCTGGCATTGTC	
cspA-F	TGCACGTAACCAGTATCGCA	A964_2021
cspA-R	GCACCGAGTTTAACGGCATC	

### 2.9. Statistical analysis

The results were presented as mean ± standard deviation (SD). *P* < 0.05 (*) was considered as being significant, and 0.05 < *P* < 0.01 (^**^) was considered highly significant. These date were analyzed by one-way analysis of variance (ANOVA). Statistical analysis was performed in SPSS (SPSS Inc., Chicago, IL, USA), and graphs were generated using GraphPad Prism 8.0 (GraphPad Software, Inc., San Diego, CA, USA).

## 3. Results

### 3.1. Construction of *SaeRS* deletion mutant strain and the complementary strain

To explore the role of *SaeRS* gene cluster in *S. agalactiae*, the mutant was constructed with deletion of a 710 bp fragment (Figure 1B). The *SaeRS* deletion strain was observed by PCR using the primers, including Sae-up-F, Sae-down-R, and Sae-F/R (Table 2), generating amplicons of 3,834 and 3,127 bp for the Δ*SaeRS* mutant strain and THN0901 wild strain, respectively (Figure 1B). DNA sequencing confirmed that the desired *SaeRS* gene cluster deletion had occurred by homologous recombination. The complementary strain CΔ*SaeRS* was also verified by PCR and DNA sequencing. The RT-qPCR analysis showed that the relative mRNA expression levels of *SaeRS* genes in the Δ*SaeRS* was not expressed at all, and the CΔ*SaeRS* strain was recovered compared with the wild strain THN0901 (*p* < 0.05) (Figure 1C). However, *SaeRS* gene deletion had little effect on the expression levels of upstream and downstream genes (Figure 1C). The results indicated that the Δ*SaeRS* mutant and complementary CΔ*SaeRS* strain was successfully constructed.

### 3.2. Growth curve

**FIGURE 2:**
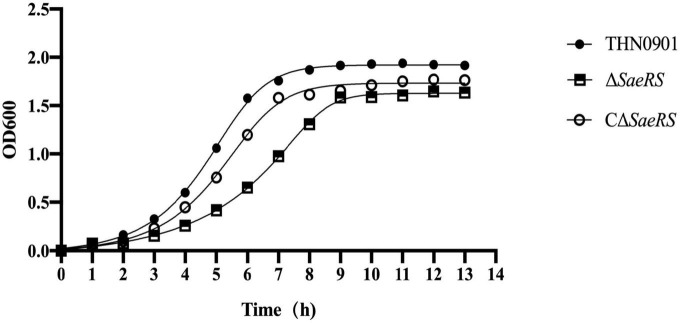
Growth curve of *S. agalactiae* THN0901, Δ*SaeRS*, and CΔ*SaeRS* strains in brain heart infusion (BHI) medium. The growth curve was checked spectrophotometrically at 600 nm.

According to the growth curves in BHI, there were some differences in the growth of the *S. agalactiae* THN0901, Δ*SaeRS*, and CΔ*SaeRS* strains under the same culture conditions. The growth rates of the Δ*SaeRS* and the THN0901 strains were significantly different. The logarithmic growth phase of the Δ*SaeRS* strain was delayed, and the time to reach the plateau phase was 2 h longer than the THN0901 strain. Also, the OD_600_ [1OD = log10 (1/trans), where trans is the transmittance *T* value of the test substance] of the THN0901 strain in the plateau phase was 0.333 higher than that of the Δ*SaeRS* strain, which means that the bacterial population of the mutant strain decreases in the plateau phase. The growth rate of the complementary strain CΔ*SaeRS* was observed between the wild strain THN0901 and the mutant strain Δ*SaeRS*, which implies that the growth rate of the strain is restored after complementing the *SaeRS* gene (Figure 2).

### 3.3. Biofilm formation

**FIGURE 3:**
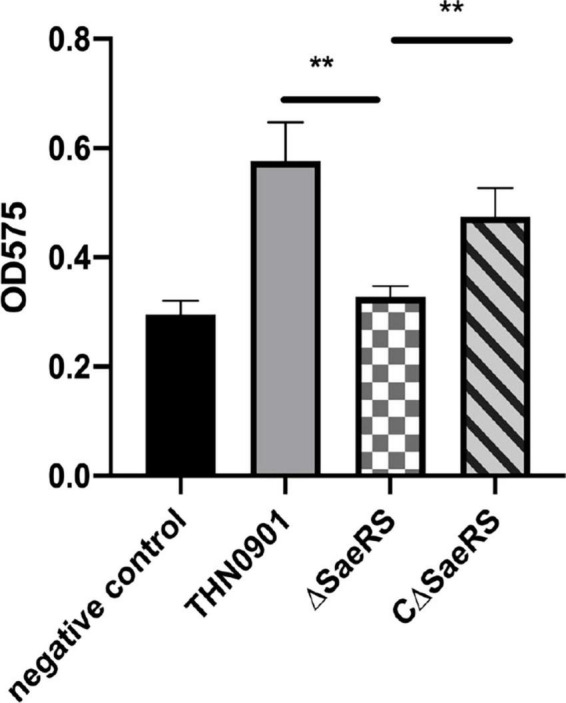
Biofilm formation of three *S. agalactiae* strains by microtiter plate assays. Each experiment was performed in triplicate, and data were presented as mean ± standard deviation (SD), *: 0.01 < *P* < 0.05, **: *P* < 0.01.

In the biofilm formation experiment, the average optical density at 575 nm (OD_575_) of *S. agalactiae* wild strains THN0901, mutant strains Δ*SaeRS*, and complementary strains CΔ*SaeRS* was 0.576 ± 0.06, 0.328 ± 0.02, and 0.475 ± 0.05, respectively, showing that the biofilm-forming ability of the Δ*SaeRS* strain was significantly lower than that of the THN0901 and the CΔ*SaeRS* strains (*P* < 0.01) (Figure 3). It means the deletion of *SaeRS* significantly reduced the ability of biofilm formation.

### 3.4. Mixed competition *in vivo* and *in vitro*

**FIGURE 4:**
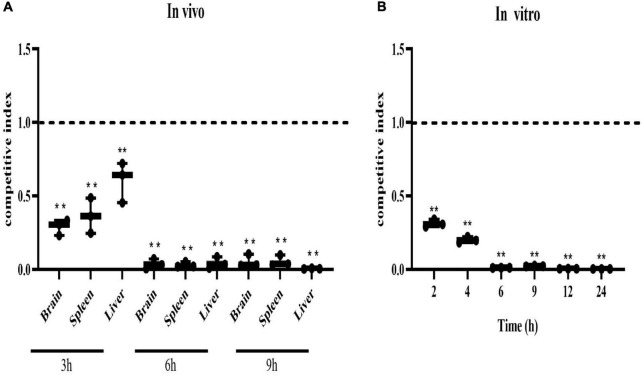
Competitive index of the Δ*SaeRS* mutant vs. total bacterial count. *In vivo*, the brain, spleen, and liver of tilapia (*Oreochromis niloticus*) infected with the mixed inoculum of the *S. agalactiae* THN0901 and Δ*SaeRS* strains were sampled and counted after 3, 6, and 12 h, respectively **(A)**. *In vitro*, the mixture of *S. agalactiae* THN0901 and Δ*SaeRS* stains was inoculated in a fresh brain heart infusion (BHI) medium, and the bacterial liquid was sampled and counted after 2, 4, 6, 9, 12, and 24 h, respectively. Samples were tested in triplicate. A competitive index of 0.5 indicated that the Δ*SaeRS* mutant strain with THN0901 strain was proliferating equally **(B)**. Each experiment was performed in triplicate, and data were presented as mean ± standard deviation (SD), *: 0.01 < *P* < 0.05, **: *P* < 0.01.

*In vivo* competition experiment, the CI values of the mutant strain Δ*SaeRS* in the brain, liver, and spleen of tilapia were significantly lower than 1 and close to 0 at 6 and 9 h, indicating that the *in vivo* colonization ability of the mutant strain Δ*SaeRS* on tilapia was significantly lower than the wild strain THN0901 (Figure 4A). The competitiveness of the mutants gradually decreased with the increase of time, and the CI value of the mutant was significantly less than 1 and close to 0 at 6 h (Figure 4B). The competitiveness of the mutant strain Δ*SaeRS* was significantly lower than that of the wild strain THN0901 both *in vivo and in vitro*.

### 3.5. Challenge test

**FIGURE 5:**
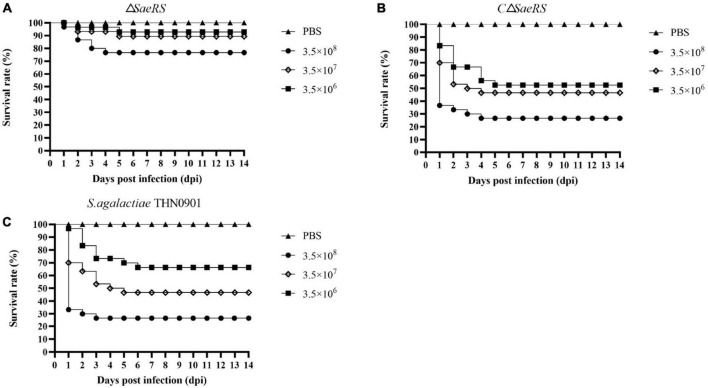
Survival curves of tilapia (*Oreochromis niloticus*) after intraperitoneal infection of *S. agalactiae* with three strains at the same dose. The mutant strain Δ*SaeRS* **(A)**. The complementary strain CΔ*SaeRS* **(B)**. The wild-type strain THN0901 **(C)**.

Tilapia were injected intraperitoneally with different concentration gradients of *S. agalactiae* THN0901, Δ*SaeRS*, and CΔ*SaeRS* strains to detect the virulence. The cumulative survival results of tilapia after the challenge are shown in Table 2, and the related survival curve results are shown in Figure 5. The time of death in tilapia was concentrated within 6 days after the challenge, and the death was stable. Under high-dose infection, the fatality rate of wild strain THN0901 and complementary strain CΔ*SaeRS* were both 73.3%, which was significantly higher than the mutant strain Δ*SaeRS* (33.3%). At low dose infection, the mortality rate of *S. agalactiae* THN0901, Δ*SaeRS*, and CΔ*SaeRS* strains to tilapia were 33.3, 46.7, and 6.7%, respectively. The virulence of the Δ*SaeRS* mutant strain was significantly lower than that of the wild strain THN0901 and complementary strain CΔ*SaeRS*.

### 3.6. Whole blood-killing assays

**FIGURE 6:**
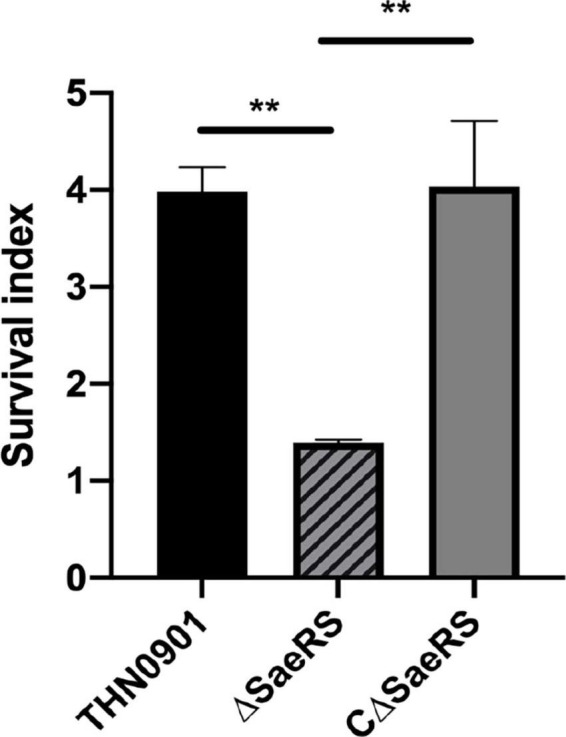
Whole blood-killing assay. The survival index was calculated. Each experiment was performed in triplicate, and data were presented as mean ± standard deviation (SD), *: 0.01 < *P* < 0.05, **: *P* < 0.01.

In this experiment, the survival index was the ratio of the colony count at the end to the initial colony count. Among them, the survival index of *S. agalactiae* wild strain THN0901 was 3.81 ± 0.207, and the Δ*SaeRS* mutant strain was 1.39 ± 0.025. The survival index of the CΔ*SaeRS* complementary strain was significantly higher than the mutant strain, which was 4.03 ± 0.551 (Figure 6). Therefore, the *SaeRS* genes are involved in survival of *S. agalactiae* in tilapia blood.

### 3.7. Virulence factor gene expression

**FIGURE 7:**
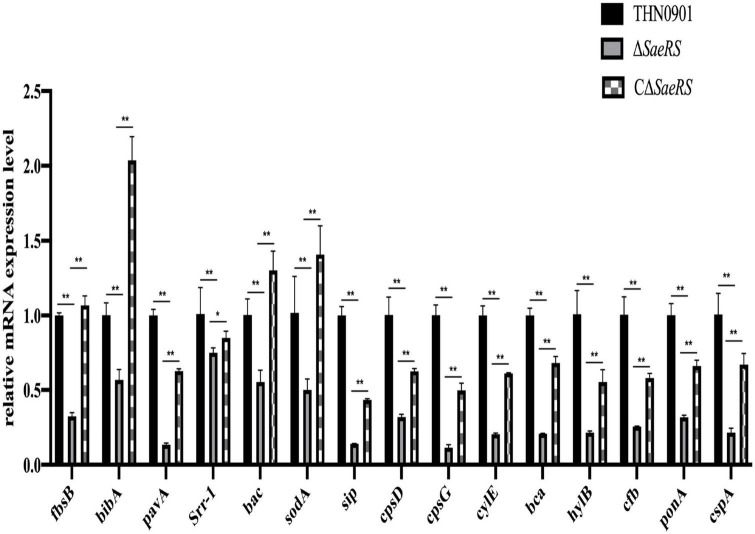
Relative messenger RNA (mRNA) expression level of virulence factors of *S. agalactiae* among three strains. Each experiment was performed in triplicate, and data were presented as mean ± standard deviation (SD), *: 0.01 < *P* < 0.05, **: *P* < 0.01.

The virulence factors related to adhesion, immune evasion, and bacterial invasion of the mutants were down-regulated compared with the wild strain THN0901 (Figure 7). Of these, the expression levels of *fbsB*, *pavA*, *sip*, *cpsG*, *cylE*, and *bca* genes in the Δ*SaeRS* mutant strain were significantly down-regulated compared with those in the wild strain THN0901 (*P* < 0.01), which decreased by 70, 90, 90, 90, 80, and 80%, respectively. After complement, the expression levels of related genes were all recovered, and most of the related virulence genes were lower than those of the wild strains. The expression levels of *fbsB*, *bibA*, *bac*, and *sodA* in the complementary strain CΔ*SaeRS* were all higher than those of the wild strain THN0901.

## 4. Discussion

Group B *Streptococcus* has at least 22 different TCSs (Faralla et al., 2014), which are almost twice as many as other streptococci. For example, there are 13 species identified in *S. pneumoniae* and *S. pyogenes* (Vega et al., 2016; Alejandro et al., 2017). GBS has a higher capability of monitoring environmental conditions and reacting to changing stimuli (Thomas and Cook, 2020). *SaeRS* is a pair of TCS, and the signal is introduced by the histidine protein kinase and transferred through a series of phosphorylation events, phosphorylating the response protein and affecting a series of physiological and biochemical reactions of bacteria (Liao et al., 2021). In genome annotation, *SaeS* and *SaeR* are considered histidine kinase and response regulator, respectively. Most of the current research on *SaeRS* of TCS focus on *S. aureus*, but the TCS of *S. agalactiae* is homologous to the well-characterized *SaeR* (48% identical) and *SaeS* (34% identical) TCS in *S. aureus* (Cook et al., 2018). To explore the function of *SaeRS* in *S. agalactiae*, the biological properties of wild strain THN0901, mutant strain Δ*SaeRS*, and complementary strain CΔ*SaeRS* were evaluated.

A previous study of *Moraxella catarrhalis* found that the inactivation of a gene (*mesR*) encoding a predicted response regulator of a TCS in *M. catarrhalis* yielded a mutant unable to grow in liquid media, which means that the response regulator is essential for growth (Joslin et al., 2015). Based on our experimental evidence, it appears that the *SaeRS* genes participate in growth regulation in *S. agalactiae*. Future research will focus on whether additional genes are upregulated in the *SaeRS* mutant to account for the observed growth defect.

*SaeRS* affects biofilm synthesis. Biofilms are heterogeneous aggregates of surface-associated microorganisms encapsulated in a self-produced polymeric matrix composed of polysaccharides, protein, and DNA, providing an ideal environment to protect bacteria from phagocytosis and harmful molecules (Kumar et al., 2017; Pang et al., 2019). In *S. aureus* Newman, a point mutation in *SaeS* resulted in the substitution of proline for leucine at amino acid 18, and the *S. aureus* Newman was unable to form a robust biofilm. RNA-Seq results showed that *Sae* regulated many genes that might affect the biofilm-forming ability of *S. aureus* Newman, such as *lytS*, *lrgA*, *atlR*, *atlA*, *arlR*, and *aaa*, they all directly or indirectly affect the formation of biofilm (Cue et al., 2015). But, deletion of *SaeRS* in *S. epidermidis* increased biofilm-forming ability, which was associated with increased environmental DNA (eDNA) release and upregulated assembly-activating protein (*Aap*) expression (Lou et al., 2011). Our experimental results are similar to those of *S. aureus* Newman. The biofilm-forming ability of the mutant strain was significantly reduced than that of the wild strain. The deletion of the *SaeRS* gene affects the expression of downstream biofilm-related genes, which leads to weakened biofilm-forming ability.

In *S. aureus*, *SaeRS* regulates bacterial survival in blood in a coagulase-dependent manner, and deletion of the *SaeRS* gene enhances the bacterial survival in human blood, which is contrary to the results of this study. Blood contains a variety of antibacterial substances, such as antibodies, phagocytes, and complements, so it has certain antibacterial functions (Ramsey, 2016). Our study showed that the survival index of the Δ*SaeRS* mutant strain in fresh tilapia blood was 1.39 ± 0.025, which was significantly lower than that of the wild strain and the complementary strain. The mutant strain had a reduced survival index through immune evasion in the blood. Some virulence factors related to immune evasion were also verified by RT-qPCR. The Cβ protein (*bac*) is one of the immunodominant components on the surface of *S. agalactiae* and can bind to the fragment crystallizable region (Fc) portion of immunoglobulin A (IgA). It may play an important role in resistance to immune defense mechanisms (Mawn et al., 1993; Schalen, 1993; Kreikemeyer and Jerlstrom, 1999; Berner et al., 2002; Das and Bishayi, 2009; Munzenmayer et al., 2016). Sip protein is a highly conserved surface protein in *S. agalactiae* with strong immunogenicity (Brodeur et al., 2000; Manning et al., 2006; Xue et al., 2010). CPS is a specific polysaccharide antigen in the outer capsule of the bacterial cell wall. The extra capsule of GBS is rich in sialic acid and can easily form oligosaccharides, which can inhibit the activation of the host’s alternative pathway of complement and significantly reduce the deposition of complement compound 3 (C3) and opsonin in cells, hindering complement-mediated opsonization and evading phagocytosis by the immune system (Marques et al., 1992). The results of q-PCR showed that the mRNA expression levels of *sip*, *bac*, *cpsD*, and *cpsG* in the mutant strain were significantly lower than those in the wild strain and recovered after complementary, which indicates that deletion of the *SaeRS* helps the immune escape of the *S. agalactiae* in blood.

In human strains of *S. agalactiae*, Cook showed through a mouse vaginal colonization model that *pbsP* and other potential genes promoted host colonization by upregulating *SaeRS* in TCS (Cook et al., 2018). Also, in *S. aureus*, inactivation of the *SaeRS* system stopped staphylococcal adhesion and lung epithelial internalization (Liang et al., 2006). The above results are consistent with the results of adhesion and colonization in this study. To explore the differences in colonization and invasion abilities between mutant strain Δ*SaeRS* and wild strain THN0901, *in vivo* competition experiment was conducted on tilapia. The colonization ability of the mutant strain in tilapia was significantly weaker than that of the wild strain, and the same results were also obtained in the *in vitro* competition. In *S. aureus*, *SaeRS*-regulated genes were associated with adhesion and invasion (Liang et al., 2006). We measured the mRNA expression of some colonization and invasion-related genes by RT-qPCR. Of these, *srr-1*, *bibA*, and *bac* genes are involved in the adhesion between *S. agalactiae* and host cells (Sundaresan et al., 2011; Souza et al., 2013; Manne et al., 2020), while *hylB* and *fbsB* genes are involved in the infection and spread of GBS in host cells (Pritchard et al., 1994; Gutekunst et al., 2004; Bobadilla et al., 2021). The decreased mRNA expression of these adhesion and invasion factors may be the reason for the *in vivo* competitive ability of mutant strain.

In *S. aureus*, mutation of *SaeRS* attenuates virulence in a mouse model of postoperative osteomyelitis, mainly due to reduced production of *S. aureus* virulence factors (Mashruwala et al., 2017). In the tilapia challenge experiment, the mortality of the mutant strain was significantly lower than that of the wild strain, which is consistent with the results of the mutants’ biofilm-forming ability and *in vitro* competition ability. The results of *S. agalactiae* colonization assays and biofilm-forming ability are closely related to bacterial virulence (Casadevall and Pirofski, 2001; Tajbakhsh et al., 2016). Attenuated virulence of mutant strain may be due to multiple effects of the above phenotypic changes, which requires further study.

In this study, the growth ability, biofilm-forming ability, *in vivo* competition ability of the *SaeRS* deletion of *S. agalactiae* THN0901 strain were lower than those of the wild strain. Therefore, *SaeRS* as a regulator of the virulence of *S. agalactiae*, plays a key role in promoting host colonization and realizing immune escape in tilapia infection. In future research, *SaeRS* can be used as the target of attenuated vaccine in production practice to enhance the immune protection effect of tilapia.

## Data availability statement

The original contributions presented in the study are included in the article/supplementary material, further inquiries can be directed to the corresponding author. The genbank ID of the primers used in the article has been supplemented in Table 3.

## Ethics statement

The animal study was reviewed and approved by the Sun Yat-sen University Animal Laboratory Center.

## Author contributions

WL conceived the study. SL, QL, and JC performed the experiments. HL analyzed the data. SL, WL, and ZL provided the reagents and technical assistance and contributed to the completion of the study. SL drafted the manuscript. AL reviewed and finalized the manuscript. All authors reviewed the results and approved the final version of the manuscript.
